# Immunoproteomics enable broad identification of new *Aspergillus fumigatus* antigens in severe equine asthma

**DOI:** 10.3389/fimmu.2024.1347164

**Published:** 2024-02-29

**Authors:** Maria-Christin Jentsch, Sabrina Lübke, Wieland Schrödl, Daniela Volke, Andor Krizsan, Ralf Hoffmann, Sarah Kaiser-Thom, Vinzenz Gerber, Eliane Marti, Bettina Wagner, Christiane L. Schnabel

**Affiliations:** ^1^ Institute of Immunology, Faculty of Veterinary Medicine, Leipzig University, Leipzig, Germany; ^2^ Institute of Bacteriology and Mycology, Faculty of Veterinary Medicine, Leipzig University, Leipzig, Germany; ^3^ Institute of Bioanalytical Chemistry, Faculty of Chemistry and Mineralogy, Centre for Biotechnology and Biomedicine, Leipzig University, Leipzig, Germany; ^4^ Swiss Institute of Equine Medicine, Department of Clinical Veterinary Medicine, Vetsuisse Faculty, University of Bern, Bern, Switzerland; ^5^ Division of Neurological Sciences, Department of Clinical Research and Veterinary Public Health, Vetsuisse Faculty, University of Bern, Bern, Switzerland; ^6^ Department of Population Medicine and Diagnostic Sciences, College of Veterinary Medicine, Cornell University, Ithaca, NY, United States

**Keywords:** immunoglobulin isotypes, proteomics, 2D Western blot, LC-MS, asthma, COPD, RAO, Heaves

## Abstract

**Introduction:**

Severe equine asthma (SEA) is a common chronic disease of adult horses with characteristic recurrent airway obstruction and similarities to neutrophilic asthma in humans. As an extrinsic stimulus, hay dust exposure is a major risk factor and induces acute exacerbation in susceptible horses. However, single inducing agents of SEA have hardly been identified on a molecular basis. *Aspergillus fumigatus* (*A. fumigatus*) is a common mold species in hay and has been described as a major provoking agent of SEA.

**Methods:**

Aiming to identify disease-relevant antigens, we analyzed *A. fumigatus* using an immunoproteomics approach on two-dimensional immunoblots of *A. fumigatus* protein probed with serum from environmentally matched asthmatic and healthy horses (n=5 pairs). *A. fumigatus* binding serum immunoglobulins (Pan-Ig), and the isotypes IgG4/7 and IgG3/5 were quantified for each protein spot and then compared between asthmatic and healthy horses.

**Results and discussion:**

For 21 out of 289 spots serum immunoglobulin (Ig) binding was different between the two groups for Pan-Ig or the isotypes. If differences were detected, Pan-Ig and IgG4/7 binding to the proteins were lower, while IgG3/5 binding was higher in asthmatic than healthy horse sera. Proteins were extracted from the 21 spots of interest and analyzed by liquid chromatography mass spectrometry. Eight prioritized proteins (candidate antigens) were expressed as recombinant proteins. Some of these have been previously described as major or minor *A. fumigatus* allergens, alongside other proteins, most with hydrolase activity. Recombinant candidate antigens were tested on 1D immunoblots to confirm their relevance as antigens by serum antibody binding. Four proteins (beta-hexosaminidase, class II aldolase/adducin domain protein, glucoamylase, peptide hydrolase B0XX53) showed different antibody binding characteristics between asthmatic and healthy horses and are likely relevant antigens in SEA. Their identification can provide the basis for innovative diagnostics, prevention, or therapeutic approaches. Additionally, a more profound understanding of SEA and its potential underlying mechanisms can be established. Elevated serum IgG3/5 antibodies correlate with T helper cell 2 responses in other equine pathologies, and the recombinant SEA antigens developed here can become instrumental in analyzing the involvement of SEA-specific T cell responses and Ig responses in future studies.

## 1 Introduction

Severe equine asthma (SEA) affects 10–20% of adult horses, which suffer from chronic cough, mucous nasal discharge, bronchospasm resulting in air flow limitation and consequently poor performance and dyspnea (1–3). SEA is the most common chronic lower airway disease of adult horses and is a major cause of economic and welfare issues (4). There is no curative treatment and the etiopathogenesis of SEA is incompletely understood. There is a consensus that dysregulated immune responses to extrinsic stimuli underlie SEA, but whether the pathogenesis is allergic is controversial (5). SEA is characterized by neutrophilic inflammation resembling severe neutrophilic asthma in humans, which is often a non-T2 endotype (6). Extrinsic stimuli, particularly hay dust, induce inflammation and symptoms in SEA-susceptible horses, whereas remission can be achieved in a dust-free environment, yet exacerbation can recur after every hay dust exposure (2, 3). Particulate matter, endotoxins, grass pollens, storage mites, and molds are contained in respirable hay dust (2, 7–9).

*Aspergillus fumigatus* (*A. fumigatus*) is a common mold species in hay and has been described as an important extrinsic stimulus of SEA. Additionally, mycological hay analyses regularly reveal fungal contamination of either good or bad quality hay (8–10). After inhalation with *A. fumigatus*, asthmatic horses showed severe symptoms of SEA in comparison to mild reactions in healthy control horses indicative of an excessive immune response to *A. fumigatus* in SEA (11–13). In these studies, mold extracts have been used, which contain various molecules and a mixture of several proteins. Specific symptom-provoking and immunogenic components of extracts are incompletely defined, and synergistic effects of proteins and other components may occur. Consequently, using mixtures for provocation studies of specific responses hampers differentiation between responses of affected and healthy individuals (14, 15).

So far, the *A. fumigatus* proteome has not been comprehensively analyzed with regard to SEA, but explored as an allergen source (7, 16–18). Analyses of antibody binding have been performed, but conflicting results regarding differences between asthmatic and healthy horse *A. fumigatus* specific serum IgE were reported when *A. fumigatus* extracts were used (7, 18–22). However, several studies used allergens defined by human allergy and detected more IgE binding to the allergens Asp f 7 and Asp f 8 in serum from asthmatic than from healthy horses (7, 20, 23, 24). Increased allergen-specific serum IgE is indicative of equine allergies, such as *Culicoides* hypersensitivity (25, 26). Nevertheless, IgE is the least abundant isotype in serum, which complicates the detection of antigen-specific IgE in the presence of competing serum IgG, and serum IgE is variable depending on season and allergen exposure (27–29). However, in allergic responses, equine IgG, particularly IgG3/5, is also induced along with IgE and can help identify immunogenic proteins as relevant antigens or allergens (29, 30). Supporting the analysis of IgG isotypes, Asp f 8- or Asp f 7-specific serum IgG were higher in asthmatic compared to healthy horses in two independent studies (7, 31).

In horses, IgG3/5 is associated with T helper 2 (Th2) responses against extracellular pathogens (32). In contrast, IgG4/7, another abundant serum isotype in horses, dominates against intracellular pathogens after exposure to viruses suggesting association with simultaneously detected T helper 1 cells (Th1) (27, 33, 34). Both, excessive Th2 or Th1 responses are discussed as pathological mechanisms for SEA and the analysis of related Ig isotypes might support the analysis of this aspect of the SEA pathogenesis (5).

We hypothesized that adaptive immune responses against a subset of the *A. fumigatus* proteins, namely relevant antigens, differ between asthmatic and healthy horses and accordingly, specific antibodies binding these antigens would differ between asthmatic and healthy horse sera, regarding their quantity and/or isotypes. As it is uncertain if SEA is an allergy, we focused on IgG, not IgE detection here. We aimed to identify relevant antigens in *A. fumigatus* protein by quantitative comparison of the antibody binding of all immunoglobulins (Pan-Ig), and Th1-associated IgG4/7, or Th2-associated IgG3/5 from serum of asthmatic – and healthy control horses. To this end, we used two-dimensional (2D) immunoblots to define differences between serum Ig binding to single *A. fumigatus* protein spots. Proteins from spots, which were differentially recognized by asthmatic and healthy horse serum Ig, were identified by mass spectrometry coupled on-line to reversed-phase chromatography (LC-MS). The candidate antigens were expressed recombinantly, and differences in serum Ig binding of asthmatic vs. healthy horse serum to the antigens were confirmed by sodium dodecyl sulfate polyacrylamide gel electrophoresis (SDS-PAGE) followed by immunoblots.

## 2 Materials and methods

### 2.1 Horse sera

Serum samples of five pairs of asthmatic and healthy control horses acquired during experiments described by Kaiser-Thom et al., under the animal experiment permission number BE110/16+ with informed consent from the horse owners, were used. Individuals were categorized and selected based on HOARSI (horse owner assessed respiratory signs index, and clinical signs (35, 36). Horses were considered asthmatic with a HOARSI of 4/4 and the presence of clinical signs of asthma (dyspnea, cough, and/or mucous nasal discharge, score 3/3) and as healthy controls with the lowest HOARSI (1/4) and absence of clinical signs (score 0/3). The endoparasitic burden was determined and considered low for each horse due to counts between 0 – 150 eggs per gram feces (McMaster method) resulting in a median of 0 for each group. The horses among pairs were matched by environment (barn, housing, and management, **Table 1**) to promote similar hay dust and *Aspergillus spp.* exposure (8–10). Samples were taken between June and October 2017. These serum samples were stored at -80°C for 5 years and kept at 4°C during the experimental series, over three months. In addition, aliquots of the same samples were used to detect antibody binding against mite proteins on immunoblots (37).

**Table 1:** Horse sera from environmentally matched^1^ asthmatic and healthy horses categorized by HOARSI and clinical examination.

Barn	Group	Sex	Breed	Age (years)	Clinical score^2^	HOARSI^3^
A	Asthmatic	Mare	Appaloosa-Quarter-Mix	20	3	4
A	Healthy	Gelding	Franches-Montagnes	18	0	1
B	Asthmatic	Gelding	Pony-Mix	20	3	4
B	Healthy	Mare	Swiss-Warmblood	13	0	1
C	Asthmatic	Gelding	Pony-Mix	28	3	4
C	Healthy	Gelding	Arabian	25	0	1
D	Asthmatic	Mare	Haflinger	20	3	4
D	Healthy	Mare	Haflinger	8	0	1
E	Asthmatic	Gelding	Criollo	18	3	4
E	Healthy	Gelding	Criollo	18	0	1

^1^Horses from one barn shared the same environmental exposure as they were kept in the same stable, fed on the same hay batches, and shared one pasture. ^2^Clinical scores for respiratory signs like dyspnea at rest, cough, and severity of mucous nasal discharge (range 0 – 3), ^3^HOARSI horse owner assessed respiratory signs index [range 1–4, (36)].

### 2.2 *Aspergillus fumigatus* protein preparation

*Aspergillus fumigatus* strain CBS 144.89 (CEA10), provided by Dr. Olaf Kniemeyer, Leibniz Institute for Natural Product Research and Infection Biology, Hans Knöll Institute, Jena, Germany was used. *A. fumigatus* spores were seeded on semi-solid growth medium (1% mycological peptone (ThermoFisher Scientific, Waltham, MA, USA), potassium phosphate buffer, pH 7.0 [3.4 mM KH_2_PO_4_, 5.75 mM K_2_HPO_4_, 0.06% thiamin, 2 mM MgCl_2_, 100 µg/ml chloramphenicol, gentamycin, chlortetracycline and 20% (w/v) Kolliphor® P 407, Sigma-Aldrich, St. Louis, MO, USA)] and grown for 1.5 days at 37°C in the dark until dense mycelia with dark spore formation were visible. *A. fumigatus* was harvested by cooling the culture to 4°C, the addition of cold phosphate-buffered saline (PBS) with 0.03% Tween-80, centrifugation for 30 min (2750 x g, 4°C), followed by three washes in 250 mM Sucrose (Sigma-Aldrich). The resulting *A. fumigatus* mycelia pellet was stored at -80°C until lysis. Lysis was performed in pre-rehydration buffer (7 M urea, 2 M thiourea, 4% 3-[(3-Cholamidpropyl)-dimethylammonio]-1-propansulfonat (CHAPS), Carl Roth, Karlsruhe, Germany) with sonication (Branson 450 Digital Sonifier) in 10 pulses of 10 sec (20 sec breaks), amplitude 35%, on ice. The lysate was recovered after centrifugation (9574 x g, 4°C, 10 min) and filtered (0.45 µm). *A. fumigatus* protein was precipitated from the lysate with ice-cold acetone (Carl Roth). The precipitated total protein (*A. fumigatus* TP) was re-suspended in a pre-rehydration buffer, and the protein concentration was determined with the Bradford method using ROTI®Quant (Carl Roth) and albumin standard (ThermoFisher) (38). One homogenous batch of *A. fumigatus* TP solution was frozen in aliquots at -80°C until use for 2D SDS-PAGE.

### 2.3 *A. fumigatus* protein separation and immunodetection

#### 2.3.1 Two-dimensional PAGE (2D) and Western blot

IPG BlueStrips (pH 3-10 NL 7 cm, Serva, Heidelberg, Germany) were incubated with 100 µg *A. fumigatus* TP in 130 µl pre-rehydration buffer with 50 mM DTT (dithiothreitol, Carl Roth), 1% (v/v) 100x BioLyte® 3/10 Ampholyte (Bio-Rad, Hercules, CA, USA), 0.001% (w/v) bromophenol blue (Sigma-Aldrich) at room temperature (rt) for six hours. Then, isoelectric focusing of the *A. fumigatus* TP on the strips was performed in a Bio-Rad Protean IEF System, with the following steps: active rehydration at 50 V for 6 h, two conditioning steps at 150 V and 300 V rapid ramp for 1 h respectively, two voltage ramping at 1000 V for 1 h and 3000 V for 2 h with linear ramp, final focusing at 3000 V for 2 h rapid ramp, and a hold at 500 V for up to 12 h. After isoelectric focusing, equilibration was performed in 6 M urea, 20% glycerol, 2% SDS, 50 mM Tris/HCl, pH 8.8, and 56 mM DTT (Carl Roth), followed by 270 mM iodoacetamide (Sigma-Aldrich) in the same buffer with agitation of 2 rpm at rt for 15 min each.

SDS gels were prepared [1 M Tris (Carl Roth), 0.1% SDS, pH 8.45, 0.5% 2,2,2-trichloroethanol (TCE, Sigma-Aldrich), 0.3% ammonium persulfate (APS), 0.03% tetramethylethylenediamine (TEMED, Merck)] with two layers of a separation (16% acrylamide, 2% bis-acrylamide, 10% glycerol, Carl Roth) and spacer part (10% acrylamide, 0.6% bis-acrylamide) in a Mini-PROTEAN® gel casting system (Bio-Rad). Focused, equilibrated *A. fumigatus* IPG BlueStrips were placed on the SDS gels covered with warm 0.5% agarose with 0.1% bromophenol blue in Tris-tricine running buffer (0.1 M Tris, 0.1 M Tricine (N-[Tris(hydroxymethyl)methyl]-glycine, Serva), 0.1% (w/v) SDS, pH 8.25). PAGE was performed using Tris-tricine running buffer (cathode) and anode buffer (0.2 M Tris, pH 8.9) in a Mini-PROTEAN Tetra Cell (Bio-Rad) with 150 W, 125 V and constant 40 mA/gel for 3 h. Activation of TCE in SDS gel to visualize *A. fumigatus* proteins by tryptophan fluorescence (TF, **Table 2**) was achieved by 1 min exposure to 300 nm UV light (ChemiDoc MP, Bio-Rad) and images of the *A. fumigatus* TP spot pattern in the gel were acquired immediately after (**Table 2**) (39). All following steps were performed protected from light.

Proteins from the SDS gel were transferred onto a nitrocellulose membrane (pore size 0.2 µm, Carl Roth) in a tank blot procedure (Mini Trans-Blot equipment (Bio-Rad) constant 300 mA, 30 min, 150 W) in transfer buffer (25 mM Tris base, 192 mM glycine (Serva), 20% (v/v) ethanol). Control images (ChemiDoc MP, Bio-Rad) of transferred proteins to nitrocellulose membrane were taken (**Table 2**). The membranes were then blocked with 1x BlueBlock PF blocking buffer (Serva) at rt with gentle agitation (2 rotations per minute, rpm), for 1 h.

**Table 2:** Antibodies, fluorochromes, and imaging specifications for 2D immunoblots.

Experimental series	Detection	Detection antibody	Reference or source	Secondary antibody	Excitation	Detection channel (filter)	Exposure time
1, 2	*A. fumigatus* TP^a^	(Tryptophan fluorescence, TF)	(39)	–	302 nm	UV (590/110 nm)	6 sec
1	Pan-Ig	goat-anti-horse Ig (H+L)- Cy3	Jackson Immuno Research (JIR) 108-165-003	–	520-545 nm	Cy3 (605/50 nm)	5 sec
	IgG3/5	mouse-anti-horse-IgG3/5 clone 586	(40, 41)	Goat-anti-mouse AlexaFluor® 647 (JIR 115-605-146)	625-650 nm	Cy5 (695/55 nm)	2 sec
2	IgG4/7	mouse-anti-horse-IgG4/7 clone CVS39	(42, 43)	Goat-anti-mouse AlexaFluor® 647 (JIR)	625-650 nm	Cy5 (695/55 nm)	2 sec

a *A. fumigatus* TP (total) protein precipitated from lyzed *Aspergillus fumigatus* mycelium.

#### 2.3.2 Immunodetection

The membranes were incubated with horse sera diluted 1:250 in 1x BlueBlock PF (2 rpm, 4°C, in the dark, overnight (o.n.), washed three times with tris buffered saline (TBS, 20 mM Tris, 150 mM NaCl), 0.05% Tween 20 (TBS-T) for 5 min each (serum dilution optimization in **Supplementary Figure 1**). Detection antibody (**Table 2**) incubation with mouse-anti-horse-IgG3/5 clone 586 (series 1, IgG3/5) or mouse-anti-horse-IgG4/7 clone CVS39 (series 2, IgG4/7) diluted in 1x BlueBlock PF for 1 h (rt, 2 rpm) followed (40, 43). After additional washing, goat-anti-mouse AlexaFluor® 647 (Jackson ImmunoResearch JIR, Dianova, Hamburg, Germany), in series 1 (IgG3/5) combined with goat-anti-horse Ig (H+L)-Cy3 (Pan-Ig) (**Table 2**) were incubated on the membrane for 1 h (rt, 2 rpm). The antibodies mouse-anti-horse-IgG3/5 clone 586 and mouse-anti-horse-IgG4/7 clone CVS39 were provided by the Wagner laboratory, Department of Population Medicine and Diagnostic Sciences, College of Veterinary Medicine, Cornell University, Ithaca, NY, USA. Finally, the membranes were washed again, and stored in dH_2_O, at 4°C for max. 2 h before imaging (**Table 2**). Immunodetection and *A. fumigatus* TP TF were recorded with the ChemiDoc MP device (Bio-Rad, **Table 2**).

### 2.4 Data curation and statistical analyses

#### 2.4.1 Data analysis of serum Ig binding on 2D immunoblots

Images taken with the ChemiDoc MP device were exported via Image Lab 6.0.1 (Bio-Rad) for analysis and further processed with Delta2D 4.8 software (DECODON, Greifswald, Germany). Protein spot patterns of all gel and membrane images were matched by exact warping, and a fused image (all gel and protein membrane images) was created. In total, 339 spots were identified after the exclusion of artefacts (e. g. gel edges) on the fused gel image and transferred to all membrane images. Raw fluorescent intensity volumes of protein (TF) and immunodetection (Cy3, Cy5) were exported to Excel.

Consistent protein transfer was confirmed by comparisons of 10 block-randomized groups of membranes (images after immunoblot transfer). Similar TF intensities were determined by ratios of the detected raw fluorescent intensity volumes per spot compared between all membranes. If more than 15% of the ratios were above 2.0 the spots were excluded, leaving 289 spots with constant protein transfer to all membranes for further analysis.

#### 2.4.2 Statistical analysis for group comparisons

Pan-Ig, IgG4/7, and IgG3/5 detection as a measure of Ig binding were exported as raw fluorescent intensity volumes (FI vol), log-transformed generating log FI vol, and further analyzed separately by isotype with GraphPad PRISM v9 software (GraphPad Software, La Jolla, CA, USA). The log FI vol were normally distributed and group comparisons (asthmatic horse vs. healthy horse sera) were performed for each spot by Two-way ANOVA with paired Fisher´s LSD test (confidence interval of 0.01). Different Ig binding of spots with significant group differences according to the ANOVA were confirmed with paired and unpaired t-tests and considered relevant if both yielded *p*<0.05.

The effects of environmental matching (**Table 1**) on Ig binding patterns were analyzed in Delta2D using hierarchical clustering with sample tree selection, Euclidean Distance as metric, and clustering with complete linkage.

### 2.5 Protein identification

*A. fumigatus* TP was separated by 2D PAGE as described above, without TCE addition, and followed by Coomassie Brilliant Blue G250 staining for protein visualization to excise protein spots from the gels in two independent experiments (44). Proteins were digested in the gel spots as previously described (45). Briefly, gel spots from the 2D PAGE or bands from SDS-PAGE of the recombinant proteins were excised with the ExQuest™ Spot Cutter (Bio-Rad) and transferred into a 96 well plate (ThermoFisher). Gel pieces were washed three times (5 min, 100 µl, 30% (v/v) acetonitrile in 50 mmol/l ammonium bicarbonate), dehydrated with acetonitrile (5 min, 100 µl), rehydrated with a mixture of 2 µl trypsin solution (50 ng/µl in 3 mM aqueous ammonium bicarbonate, Serva) and 18 µl of 3 mM aqueous ammonium bicarbonate. After incubation (37°C, 4 h), supernatants were transferred to new 0.5 ml reaction tubes. The remaining gel pieces were washed once with 60% (v/v) aqueous acetonitrile containing 0.1% (v/v) formic acid and acetonitrile (20 µl per tube, rt, 5 min). Supernatants were transferred to the corresponding reaction tube and dried (60°C, 1 h) in a vacuum concentrator 5301 (Eppendorf Vertrieb Deutschland GmbH, Hamburg, Germany). The dried digests were dissolved in a mixture of 1.5 µl of acetonitrile containing 0.1% (v/v) formic acid (eluent B) and 48.5 µl of 0.1% aqueous formic acid (eluent A) and analyzed on a nanoACQUITY Ultra Performance LC™ (Waters Corp., Manchester, UK) system coupled online to a Q-TOF SYNAPT G2-Si instrument (Waters Corp., UK). Peptides were trapped on a nanoACQUITY Symmetry C_18_-column (internal diameter (ID) 180 µm, length 2 cm, particle diameter 5 µm) at a flow rate of 5 µl/min (3% eluent B, 6 min) and separated on a C_18_-BEH 130 column (ID 75 µm, length 10 cm, particle diameter 1.7 µm; 35°C) using a flow rate of 0.3 µl/min and a linear gradient from 3% to 40% eluent B in 18.5 min. The nanoESI source was equipped with a PicoTip Emmitter (New Objective, Littleton, US) at a spray voltage of 3 kV, sampling cone was 30 V, source offset 80 V, source temperature 100°C, cone gas flow 20 l/h, and nanoflow gas pressure 0.2 bar. Mass spectra were recorded in positive ion mode using a high-definition data-dependent acquisition approach (HD-DDA) for the six most intense signals (top 6 ions).

LC-MS/MS raw files were processed with Mascot Distiller (Version 2.8.1.0; Matrix Science Ltd, UK) and searched with the Mascot search engine (Version 2.7.0; Matrix Science Ltd., UK) using the following parameters: UniProtKB *Aspergillus fumigatus* CBS 144.89 (Database (Oxford) 01.09.2021; 9942 sequences), enzyme trypsin, 2 miss cleavage sides, as fixed modification cysteine carbamidomethylation (+57.022 Da), as variable modification methionine oxidation (+15.9949 Da), 20 ppm peptide tolerance and 0.08 Da fragment tolerance. Spectral Library files were created from the Mascot search engine results (.dat files) in Skyline (https://skyline.ms/project/home/software/Skyline/begin.view) and the.raw files were uploaded to the skyline document for further inspection. Data can be accessed on ProteomXchange PXD045869 or Panorama https://panoramaweb.org/Asp-f-in-SEA.url. Proteins identified by at least three peptides and a peptide score ≥ 50 were considered as confident. Confidently identified proteins were further categorized by (i) description as allergens, (ii) verification in duplicate LC-MS analysis, and/or appearance within at least 10% of the spots of interest.

### 2.6 *A. fumigatus* culture for ribonucleic acid isolation and complementary deoxyribonucleic acid synthesis

*A. fumigatus* strain CBS 144.89 was grown in liquid culture media (1% mycological peptone (ThermoFisher), potassium phosphate buffer, pH 7.0 (3.4 mM KH_2_PO_4_, 5.75 mM K_2_HPO_4_, Sigma-Aldrich), 0.06% thiamin (Sigma-Aldrich), 2 mM MgCl_2_ (Sigma-Aldrich), 100 µg/ml chloramphenicol, gentamycin, chlortetracycline) and cultured for 24 h at 37°C. Mycelium was harvested by centrifugation (2750 x g, 4°C, 30 min), and washing in PBS. The dried pellet was stored at -80°C until ribonucleic acid (RNA) isolation.

*A. fumigatus* cultured for RNA isolation was frozen with liquid nitrogen, ground in a mortar, and treated with RNA-Solv® Reagent (Omega Bio-tek, Norcross, GA, USA) according to the manufacturer’s instructions (including additional steps for plant samples). RNA was re-suspended in RNase-free water and concentration was determined with NanoDrop (ThermoScientific). The following cDNA synthesis was performed with 2 µg *A. fumigatus* RNA and High-Capacity complementary deoxyribonucleic acid (cDNA) Reverse Transcription Kit (ThermoFisher) according to the manufacturer’s protocol, except using Oligo-dT primers (ThermoFisher) on MJ Research PTC 200 Peltier Thermal Cycler (Bio-Rad) instrument with the following cycles: 25°C for 10 min, 37°C for 120 min, 85°C for 5 min, and stored at 4°C.

### 2.7 Cloning and recombinant protein expression of candidate antigens

Primers suitable for the expression vector pET28a (+) (Sigma-Aldrich) were designed and amplification conditions were established (**Table 3**; **Supplementary Table 1**). Phusion™ High-Fidelity DNA Polymerase (ThermoFisher) was used to amplify genes of interest by polymerase chain reaction (PCR) using 1 µl cDNA on an MJ Research PTC 200 Peltier Thermal Cycler (Bio-Rad) instrument with the following steps – one cycle for initial denaturation (98°C, 60 sec), 40 cycles for denaturation (98°C, 15 sec), annealing (target gene specific temperature, **Table 3**, **Supplementary Table 1**, 30 sec) and elongation (72°C, target gene specific time, **Table 3**, **Supplementary Table 1**) and one cycle for final elongation (72°C, 10 min). Amplified products were stored at 4°C. PCR products were purified using NucleoSpin® PCR (Macherey-Nagel). The vector pET28a (+) was propagated in chemically competent *Escherichia coli* (*E. coli*) strain DH5α (New England Biolabs (NEB), Ipswich, MA, USA) and purified with QIAprep® Spin Miniprep Kit (Qiagen, Venlo, Netherlands). PCR products and the vector were digested with the restriction enzymes in CutSmart buffer (NEB) at 37°C for 1h 40 min (**Table 3**): BamHI-HF (R3136S), HindIII-HF (R3104S), NdeI (R0111S), NotI (R3189S), and XhoI (R0146S). Additionally, the digested vector was dephosphorylated using Quick CIP (M0525S, NEB) according to the manufacturer’s instructions. The digested products and vector were purified with NucleoSpin® PCR (Macherey-Nagel) and ligated with Quick Ligation™ Kit (NEB) according to the manufacturer’s protocol.

**Table 3:** Target gene amplifications with primer, and PCR conditions.

Target protein	Uniprot	Forward primer (restriction site) ^a–e^	Reverse primer (restriction site) ^a–e^	Annealing temperature	Elongation time
Allergens (allergome reference)					
Alkaline protease 1 (Asp f 13)	B0Y708	*CGCTAAGCATATGCCTGTCCAGGAAACTCGTC* ^a^	*GCGTTACCTCGAGAGCATTGCCATTGTAGGCAAG* ^b^	55°C	40 sec
Catalase (Asp f Catalase)	B0Y0G0	*GCTGTTGCGGCCGCGTATGTCCCTATATGACCGGC* ^c^	*GCTTTACTCGAGGTGATCCACGGGAAACCGG* ^b^	56°C	80 sec
Dipeptidyl-peptidase 5 (DPPV)	B0XRV0	*CGGCATCATATGGGAGCTTTCCGCTGGCT* ^a^	*GCAATAGCGGCCGCGTTATAATTCACAACCGGGACAA* ^c^	54°C	80 sec
Not described as allergens					
Beta-hexosaminidase	B0Y9W3	*AATGTTGCGGCCGCATGCTCATCTCCAGCATCTGC* ^c^	*GCTTTACTCGAGCGCGACGGCACTTTGGTC* ^b^	56°C	80 sec
Class II aldolase/adducin domain protein	B0XWR5	*CGCGTTCATATGCGTGCTTCTTTCATTCTCTTCTC* ^a^	*GTATATGCGGCCGCCCAGCCGCTCTCCAAGC* ^c^	55°C	40 sec
Glucoamylase	B0XSV7	*GCTTTACATATGGCTCCTCAGTTATCCGCTC* ^a^	*CGTTCACTCGAGCCGCCAAGTATCATTCTCGG* ^b^	55°C	80 sec
Peptide hydrolase B0XX53	B0XX53	*CGCTCACATATGGTCACCATGAAGCTGCTCTAC* ^a^	*GCTTTACTCGAGCTGCTCAACCCGGTCCTTG* ^b^	56°C	80 sec
Probable Xaa-Pro aminopeptidase pepP	B0XW47	*CGCCTTCATATGATGGCCGCGGTAGATGCAAT* ^a^	*GCGAATGCGGCCGCGGATGCAGCTAGTCTCTCTAG* ^c^	56°C	80 sec

Restriction sites for (underlined): ^a^Ndel, ^b^Xhol, ^c^Notl, ^d^HindIII, ^e^BamHl.

Plasmids with target genes as insert were propagated in chemically competent *E. coli* strain DH5α (NEB) using the heat shock method and plated on lysogeny broth (LB)-agar plates (1% (w/v) tryptone, 0.5% (w/v) yeast extract, 171 mM NaCl, 1.5% (w/v) agar-agar (all chemicals used from Carl Roth), pH7), containing 30 µg/ml kanamycin (AppliChem), incubated at 37°C o.n. Colonies were picked and controlled for inserts of the correct length using DreamTaq DNA Polymerase (ThermoFisher), cultured again in liquid LB-medium, containing 30 µg/ml kanamycin at 37°C o.n., and used for plasmid isolation with QIAprep® Spin Miniprep Kit (Qiagen). Isolated plasmids with target insert were transformed into *E. coli* strain Rosetta pLysS (Sigma-Aldrich), which was performed like the previous transformation process, but cultured on LB-agar plates containing 15 µg/ml kanamycin and 34 µg/ml chloramphenicol (Euroclone, Pero, Italy).

To express recombinant proteins, *E. coli* strain Rosetta pLysS colonies with the insert of interest were picked from LB-agar plates, pre-cultured in LB-medium containing 15 µg/ml kanamycin and 34 µg/ml chloramphenicol at 37°C, 200 rpm o.n., expanded in fresh medium to OD_600_ 0.4–0.6, and then 1 mM isopropylthiogalactoside (IPTG, Sigma-Aldrich) was added to the culture for most recombinant targets. For dipeptidyl-peptidase V 0.3 mM IPTG and glucoamylase 2 mM IPTG were used after optimization. Bacteria were harvested after 4 h incubation with IPTG, pelleted by centrifugation (10,000 x g, 2 min), and stored at -80°C.

For SDS-PAGE transformed bacterial pellets with recombinant (*r*) proteins were re-suspended in Lämmli buffer (0.125 M Tris-HCl pH 6.75, 20% glycerol, 2.5% SDS, 10% 2-β-mercaptoethanol (Sigma-Aldrich), 0.05% bromophenol blue) and boiled at 95°C for 15 min.

The recombinant proteins’ identities were confirmed using SDS-PAGE and immunoblots indicating over-expression after IPTG induction of the expected molecular weights, immunoblots (performed as described for 2D PAGE, but gels with a stacking (5.7%) and separation (12%) part) detected with Anti-His antibody (652501, BioLegend), and goat-anti-mouse AlexaFluor® 647(Jackson ImmunoResearch). LC-MS confirmation of the targeted proteins’ peptide in the respective bands cut out from Coomassie-stained SDS gels (as described for *A. fumigatus* protein spots) and LC-MS were performed as described above.

### 2.8 Immunoreactivity of serum Ig with *A. fumigatus* recombinant proteins on 1D immunoblots

The *A. fumigatus* recombinant (*r*) proteins were expressed in *E. coli* Rosetta with IPTG induction as described above and bacterial cell pellets frozen at -80°C, resuspended in PBS or pre-rehydration buffer, and homogenized in precellys tubes (Bertin Technologies, Montigny-le-Bretonneux, France) with quartz sand (0.4 – 0.8 mm, Carl Roth) with a Precellys 24 Homogenzier (6800 rpm 3 x 30 sec twice). After centrifugation, supernatants were stored at -80°C. The acetone precipitation of *r* proteins was performed as described for *A. fumigatus* TP, to reduce bacterial product background in one-dimensional (1D) immunoblot analyses.

The acetone precipitated *r* proteins were used to test their immunoreactivity with the serum samples used before (**Table 1**). Precipitated bacterial lysates with overexpressed *r* proteins were separated by (1D) PAGE. As controls, acetone precipitated bacterial lysate before IPTG induction (negative control) and *r*Asp f 1 pure protein (positive control, kindly provided by Dr. Claudio Rhyner, SIAF, Davos, Switzerland) were included. PAGE, blotting, serum, and detection antibodies (Pan-Ig, IgG4/7, IgG3/5, goat-anti-mouse AlexaFluor® 647) were applied as described above for 2D immunoblots (**Table 2**).

#### 2.8.1 Data analysis of serum Ig binding on 1D immunoblots

Images of 1D immunoblots were taken by ChemiDoc MP device and analyzed with Image Lab 6.0.1 (Bio-Rad). Protein bands were marked as rectangular regions of interest on the TF image representing the recombinant proteins and copied onto the images with corresponding immunodetection signals (Cy3, Cy5). The volume table was exported to Excel where the adjusted volumes were normalized by dividing Cy3– or Cy5-fluorescence by TF fluorescence to yield ratios of serum Ig binding: *r* protein. Normalized data were further analyzed with GraphPad PRISM v9 software (GraphPad Software) using Wilcoxon tests to compare asthmatic and healthy horse serum Ig binding to the single recombinant proteins.

## 3 Results

### 3.1 Different antibody binding to *A. fumigatus* protein spots between asthmatic and healthy control horse sera

**Figure 1:**
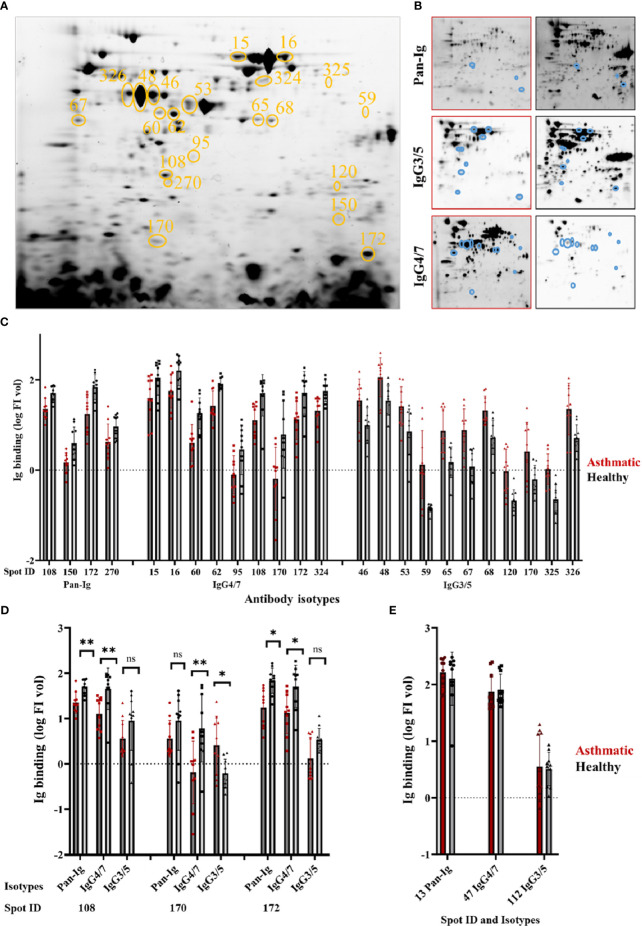
Distinct *A*. *fumigatus* protein spots yield different immunoglobulin binding by asthmatic versus healthy horse sera. *Aspergillus fumigatus *(*A. fumigatus*) protein was separated by isoelectric focusing and SDS-PAGE resulting in **(A)** a characteristic *A*. *fumigatus* protein spot pattern (331 spots) visualized by tryptophan fluorescence (TF). Spots yielding different serum Ig binding with asthmatic vs. healthy horse sera are circled in orange (n=21) and numbers indicate the spot ID. The membranes were probed with sera from asthmatic or healthy horses (n=5 pairs), and immunodetections of all bound antibodies (Pan-Ig, Cy3), IgG4/7 (Cy5), and IgG3/5 (Cy5) were quantified per protein spot as illustrated in **(B)** representative examples with asthmatic (left, red frame) and healthy (right, black frame) horse serum. Spots with group differences are indicated by blue circles. **(C)** Serum Ig binding (log FI vol) of Pan-Ig, IgG4/7, or IgG3/5 are plotted for spots, which yielded different Ig binding between asthmatic and healthy horse sera (all *p*<0.01 in ANOVA). Dots, squares, or triangles represent individual measurements’ log FI vol per spot (sera in duplicates), and bars represent group means. Asthmatic horse sera yielded lower binding of Pan-Ig, or IgG4/7 than healthy horse sera. In contrast, asthmatic horse sera yielded higher IgG3/5 binding than healthy horse sera. **(D)** Isotype comparison of Pan-Ig, IgG4/7, and IgG3/5 for shared spots 108, 170, and 172; ns, not significant, * *p*<0.05 and ** *p*<0.01 in t-tests. **(E)** Internal control spots with strong binding of respective serum isotypes but without group differences.

This study targeted the identification of immunogenic *A. fumigatus* proteins with relevance for SEA. To identify these proteins on 2D immunoblots, we compared *A. fumigatus* immunodetection by serum antibodies from asthmatic with those from healthy horses (**Table 1**). Immunodetection showed significant group differences in Ig binding with asthmatic compared to healthy horse serum for Pan-Ig, IgG4/7, and IgG3/5 with 21 of 289 *A. fumigatus* protein spots with consistent protein transfer (**Figure 1A**). Four protein spots suggested lower Pan-Ig binding (log FI vol) with sera from asthmatic compared to the healthy horses (*p*<0.01, **Figures 1B, C**). Similarly, nine protein spots showed lower IgG4/7 binding with asthmatic compared to healthy horse sera (*p*<0.01, **Figures 1B, C**). With two of the spots (108 and 172), Pan-Ig and IgG4/7 group differences of the same orientation were detected (**Figure 1D**). In contrast, eleven protein spots yielded significantly higher IgG3/5 binding with asthmatic - than with healthy horse sera (*p*<0.01, **Figures 1B, C**). Spot 170 exhibited elevated IgG3/5 binding in the presence of serum from asthmatic horses, while it displayed reduced IgG4/7 binding compared to healthy horse sera and similar Pan-Ig binding (ns, **Figure 1D**).

Individual spot patterns for each horse, but also Pan-Ig, and the isotypes IgG4/7 and IgG3/5 were detected (**Supplementary Figure 2**). Most individual sera shared Pan-Ig and Ig isotype binding to spots in the high molecular weight region (**Supplementary Figure 2**). The environmental matching of asthmatic and healthy horses as pairs (**Table 1**) was not effective in hierarchical cluster analyses of the Ig binding patterns (**Supplementary Figure 3**).

### 3.2 *A. fumigatus* proteins as candidate antigens

The 2D immunoblots of *A. fumigatus* proteins revealed different antibody binding between asthmatic and healthy horse sera with 21 *A. fumigatus* protein spots (**Figure 1**). These spots were selected to identify the proteins using LC-MS. Additionally, three control spots were selected for LC-MS as positive immunogenic controls (spot 13 for Pan-Ig, spot 47 for IgG4/7, and spot 112 for IgG3/5), which showed high antibody binding, without differentiating asthmatic or control horse sera (**Figure 1E**).

A total of 79 proteins were identified in the 21 spots of interest and three control spots (immunogenic control proteins) in the UniProtKB *Aspergillus fumigatus* CBS 144.89 database (https://panoramaweb.org/Asp-f-in-SEA.url). The proteins were ranked in four groups (1: proteins described as allergens, 2: proteins verified in duplicate LC-MS *and* appearance within 10% or more of spots of interest, 3: proteins verified in duplicate LC-MS *or* appearance within 10% or more of spots, 4: proteins not prioritized by these criteria) and the 19 proteins in the first three groups were selected as candidate antigens. Five proteins have been described as allergens, and ten proteins have hydrolase or glycolase activity (**Table 4**; **Supplementary Table 2**).

**Table 4:** *A. fumigatus* proteins of interest identified by LC-MS and ranked as candidate antigens.

Isotype with group difference on 2D immunoblots or immunogenic control (C)					Pan-Ig				IgG4/7									IgG3/5											C Pan-Ig	C IgG4/7	C IgG 3/5
Protein identified ^A^	Uni prot ID ^A^	Described allergen^B^, hydrolase (hy), glycolytic (gl) activity	Rank	Spot ID	108	150	172	270	15	16	60	62	95	108	170	172	324	46	48	53	59	65	67	68	120	170	325	326	13	47	112
Allergens																															
Alkaline protease 1	B0Y708	Asp f 13, hy	**1**																												**x**
Catalase	B0Y0G0	Asp f Catalase	**1**						**x**	**x**					**x**		**x**					**x**		**x**	**x**	**x**	**x**				
Dipeptidyl-peptidase 5	B0XRV0	DDPV, hy	**1**		**x**	**x**	**x**	**x**	**x**	**x**	**x**	**x**	**x**	**x**		**x**	**x**			**x**	**x**	**x**		**x**	**x**						
Not described as allergens																															
Beta-hexosa-minidase	B0Y9W3	hy, gl	**2**															**x**	**x**									**x**			
Class II aldolase/adducin domain protein	B0XWR5		**2**																						**x**						**x**
Gluco-amylase	B0XSV7	hy, gl	**3**														**x**	**x**												**x**	
Peptide hydrolase B0XX53	B0XX53	hy	**2**										**x**					**x**	**x**	**x**			**x**					**x**			
Probable Xaa-Pro aminopepti-dase pepP	B0XW47	hy	**2**		**x**							**x**	**x**	**x**						**x**		**x**		**x**	**x**						

^A^ *A. fumigatus* CBS 144.89, ^B^Uniprot based information.

### 3.3 Confirmation of candidate antigens

**Figure 2:**
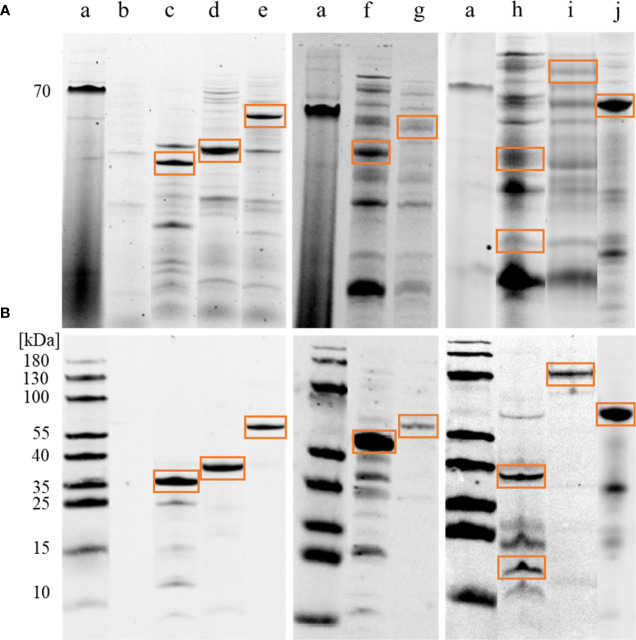
Immunoblot confirmation of *A. fumigatus* recombinant protein expression. *E*. *coli* lysates with *Aspergillus fumigatus* (*A. fumigatus*) recombinant proteins were tested for correct molecular weight and 6xHis-tags in 1D immunoblots. **(A)** Tryptophan fluorescence of proteins in the whole *E. coli* lysate with recombinantly expressed *A. fumigatus* proteins on 1D SDS-PAGE. (a) PageRuler Prestained Protein Ladder (ThermoFischer), (b) empty vector pET28a(+) after IPTG induction and acetone precipitation as negative control, (c) alkaline protease 1 (Asp f 13), 42 kDa, (d) class ll aldolase/adducin domain protein, 32 kDa, (e) peptide hydrolase B0XX53, 54 kDa, (f) probable Xaa-Pro aminopeptidase pepP, 52 kDa, (g) beta-hexosaminidase, 67 kDa, (h) catalase, 80 kDa expected weight, but separated into two bands of approximately 38 kDa and 20 kDa, (i) dipeptidyl-peptidase 5 (DPPV), 80 kDa, (j) glucoamylase, 67 kDa. **(B)** Anti-His-tag antibody followed by goat-anti-mouse AlexaFluor® 647 were used for detection (Cy5). Note that the (red) 70 kDa band of the protein ladder is visible in TF **(A)** while (blue) 180–10 kDa bands are visible in Cy5 **(B)**.

**Figure 3:**
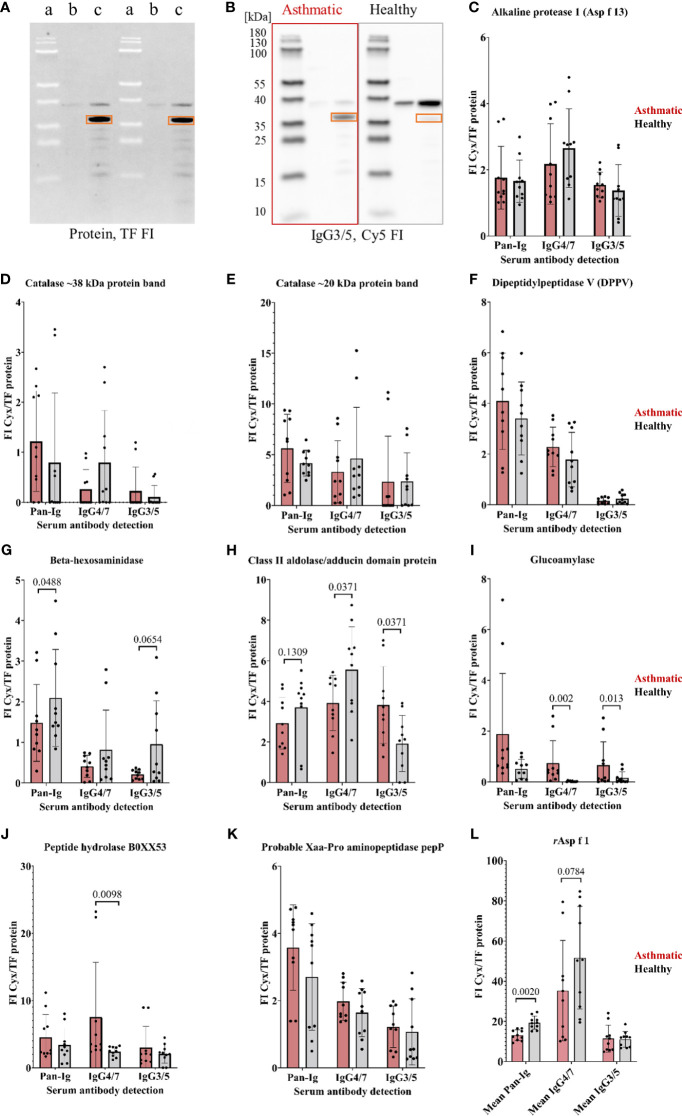
*A. fumigatus* recombinant proteins confirmed as antigens by Ig binding differences of asthmatic versus healthy horse sera. *A. fumigatus* recombinant proteins (in *E. coli* lysates) were analyzed in 1D immunoblots with asthmatic and healthy horse sera (n=5 pairs). **(A)** 1D immunoblot with tryptophan fluorescence (TF) with (a) PageRuler Prestained Protein Ladder, *E. coli* lysate (b) before IPTG induction, and (c) after IPTG induction with recombinant alkaline protease 1 (Asp f 13) framed with orange rectangles, and corresponding representative example of **(B)** immunodetection of IgG3/5 binding to alkaline protease 1 (Asp f 13) highlighted with orange rectangles after probing with sera from an asthmatic and a healthy horse; **(C–K)** Serum Ig binding was determined by normalization of the immunodetection signal (Cy3 or Cy5 fluorescence intensity, FI) to the protein signal (tryptophan fluorescence, TF FI) resulting in normalized fluorescent intensities (FI Cyx/TF protein). Significant group differences were found for beta-hexosaminidase **(G)**, class II aldolase/adducing domain protein **(H)**, glucoamylase **(I)**, and a peptide hydrolase B0XX53 **(J)** (using Wilcoxon tests, *p*<0.1); **(L)** Mean immunodetection results of three separate experiments using *r*Asp f 1 as control protein revealing significantly lower Pan-Ig binding with asthmatic compared to healthy horse sera for all immunoglobulins. Note that the protein ladder is visible by (negative) blocking effects in TF (**A**, a) and the fluorescence in Cy5 **(B)**.

Eight of the candidate antigens were sufficiently expressed as recombinant proteins in *E. coli* to verify the immunogenicity and different binding by asthmatic compared to healthy horse serum Ig. The eleven other candidates were not sufficiently expressed, or less prioritized, and not attempted here (**Supplementary Table 2**). Successful recombinant protein expression of the eight proteins was confirmed by His-tag detection and correct molecular weights on 1D immunoblots in *E. coli* lysates (**Figure 2**) and the protein identities were confirmed by LC-MS (https://panoramaweb.org/Asp-f-in-SEA.url). Catalase was separated into two bands of ~38 kDa and ~20 kDa, which were analyzed separately (**Figure 2** lane h; **Figures 3D, E**).

Immunoreactivities of the recombinant proteins were tested on 1D immunoblots (**Figures 3A, B**) by serum binding of Pan-Ig, IgG4/7, and IgG3/5, and different Ig binding of asthmatic vs. healthy horse sera confirmed four antigen candidates (**Figures 3G–J**). Beta-hexosaminidase showed lower Pan-Ig *and* IgG3/5 binding by asthmatic than healthy horse serum, but IgG4/7 binding was similar between the groups (**Figure 3G**). Class II aldolase/adducin domain protein also showed a tendency of less Pan-Ig binding by asthmatic horse serum compared to healthy individuals (**Figure 3H**). Additionally, class II aldolase/adducin domain protein yielded significantly lower IgG4/7 but higher IgG3/5 binding by asthmatic compared to healthy horse serum (**Figure 3H**). Moreover, higher IgG4/7 binding was detected for asthmatic compared to healthy horse serum for peptide hydrolase B0XX53 (**Figure 3J**) and glucoamylase (IgG4/7, IgG3/5) (**Figure 3I**). Alkaline protease 1 (Asp f 13), catalase, dipeptidyl-peptidase 5 (DPPV), and probable Xaa-Pro aminopeptidase pepP did not reveal significant group differences and were not confirmed as relevant antigens (**Figures 3C–F, K**). Recombinant Asp f 1 protein was used as positive control and revealed significant group differences with lower Pan-Ig binding by asthmatic horse serum (**Figure 3L**, averages from three separate experiments).

## 4 Discussion

2D immunoblots and LC-MS of *A. fumigatus* proteins were applied for the first time for the investigation of SEA, as it involves the analysis of proteins in an antigen source of interest without prior selection bias for known proteins. In this study, we used this bottom-up approach and quantified antibody binding with *A. fumigatus* proteins separated by 2D immunoblots using multiplexed fluorescent detection. This demonstrated different Pan-Ig, IgG4/7, and IgG3/5 binding between asthmatic and healthy horse sera to several spots, but not all *A. fumigatus* proteins. Candidate antigens were defined by these significant differences in Ig binding to *A. fumigatus* protein spots on immunoblots. The identified proteins of *A. fumigatus* spots after LC-MS were ranked, 19 candidate antigens were prioritized and eight of them were tested as recombinant proteins, of which four were confirmed as likely relevant antigens in SEA, which are all newly described with relevance for asthma.

The majority of *A. fumigatus* protein spots on the 2D immunoblots were immunogenic, as evidenced by serum Ig binding from both asthmatic and healthy horses. However, protein spots with significant group differences were revealed by quantitative discrimination and resulted in group specific binding patterns. Notably, in asthmatic horse sera an elevated IgG3/5, but reduced Pan-Ig and IgG4/7 responses compared to healthy controls were observed. Serum IgG3/5 binding predominance and decreased IgG4/7 in SEA in our study might indicate a shift to type 2 responses in severely asthmatic horses while healthy individuals predominantly produce antigen specific IgG4/7 indicative of type 1 responses (34). Even if a type 2 shift matches an allergic pathogenesis (T2 endotype), IgG3/5 detection does not prove this directly and this hypothesis remains to be confirmed. Furthermore, a major *A. fumigatus* allergen, Asp f 1, was included in the analyses here for comparison and did not yield IgG3/5 binding differences (29, 34).

Reduced IgG4/7 binding with asthmatic horse sera with certain *A. fumigatus* proteins could be based on reduced specific IgG4/7, e.g. for spots 108 and 172, and might indicate an impaired adaptive (type 1) immune response in asthmatic horses. In addition, the control protein *r*Asp f 1 also showed a tendency of reduced IgG4/7 binding in asthmatic horses´ sera when tested in 1D immunoblots which has not been observed before (18). Another explanation for reduced IgG4/7 binding is an increase in specific serum antibodies of other isotypes that compete for the same epitopes IgG4/7 would bind. This mechanism could contribute to decreased IgG4/7 but increased IgG3/5 binding with spot 170. The complexity of several isotypes’ binding in serum may have led to the fewer differences identified in the analysis of Pan-Ig compared to single IgG isotypes. Accordingly, the analysis of separate IgG isotypes appears superior over a global Ig binding analysis, also with regard to specific antigens.

Isotype patterns of 2D immunoblot spots were reflected in some *r* protein-isotype combinations. Class II aldolase/adducing domain protein and glucoamylase showed similar binding patterns for IgG3/5 with a higher binding by asthmatic compared to healthy horse serum in 2D and 1D immunoblots. With this, these proteins could support IgG3/5 binding predominance and the indication of a shift to specific type 2 responses in SEA. Globally, this was not indicated as total serum IgG3/5 was similar between healthy and asthmatic horse sera and the direct comparison of T cell responses was not possible in the present study (37).

Mycological analyses of hay consistently disclose the presence of fungal contamination in both visually satisfactory and suboptimal quality hay (8, 9). Consequently, we anticipate that the horses used in this study were exposed to a variety of different fungal contaminants and several *A. fumigatus* proteins. It needs to be considered that one well-characterised *A. fumigatus* strain, CEA10, was used in this study and its proteome might partially differ from *A. fumigatus* from hay (46).

We covered a broad range of immunoreactive proteins, which were bound by most horses’ antibodies. These are likely conserved among different *Aspergillus* strains and thus provoke consistent Ig production in horses with variable *Aspergillus* exposure. For example, catalase and beta-hexosaminidase of the strain CEA10 used here share 100% protein identity with the respective proteins from another strain (CBS 101355, catalase Q92405, beta-hexosaminidase Q4WCB5). Further investigations might consider *A. fumigatus* isolates from hay to determine additional, isolate-specific immunoreactive proteins.

*A. fumigatus* proteins have generally been described as allergens causing severe asthma in humans, or allergic airway responses in conjunction with aspergillosis. Horses do not commonly develop aspergillosis, but *A. fumigatus* exposure is common in stabled horses (47). Nevertheless, *A. fumigatus* allergens yielded higher antibody binding in asthmatic compared to healthy horse sera (7, 20, 24).

In human aspergillosis, mainly secreted proteins were found to provoke a host´s immune response matching the overrepresentation of soluble proteins in the candidate antigens here. However, extraction and 2D immunoblot procedures may favour the representation of easily soluble proteins additionally (48). Nevertheless, major allergens of *A. fumigatus* in human allergies, such as Asp f 1, were not indicated by our proteomics analyses.

From the *A. fumigatus* proteome analyzed here, eight of 19 selected proteins of interest were recombinantly expressed in *E. coli* and serologically tested on 1D immunoblots, and four proteins can be considered as new candidate antigens: Beta-hexosaminidase, class II aldolase/adducing domain protein, glucoamylase, and a peptide hydrolase B0XX53 which have not been described as *A. fumigatus* allergens. Beta-hexosaminidase and glucoamylase have catalytic activity as hydrolases and glycosidases. Beta-hexosaminidase is located in membranes, and belongs to the glycosyl hydrolase 20 family, whereas glucoamylase is part of the glycosyl hydrolase 15 family (49). Peptide hydrolase B0XX53 functions as aminopeptidase, metalloprotease, and also as hydrolase. Moreover, this protein has a metal-binding function of zinc and belongs to the peptidase M28 family with M28A as a subfamily. Interestingly, this hydrolase shows 100% identity with probable leucine aminopeptidase 2 of *A. fumigatus*, which may support relevance in *A. fumigatus* pathogenicity deducted from the similarity (UniProt Consortium). In contrast, molecular functions or involvements in biological processes of the *A. fumigatus* class II aldolase/adducing domain protein have not been characterized. In summary, these four candidate proteins might be reconsidered in further studies as they all seem relevant antigens in SEA. Therefore, the recombinant proteins can form a basis to further analyze antigen-specific responses in the pathogenesis of equine asthma and possibly in other species. Furthermore, elucidating specific antigens can contribute to early diagnostic interventions, potentially prior to the clinical manifestation of SEA.

Peptide hydrolase B0XX53 and glucoamylase showed higher IgG4/7 binding with asthmatic horse serum which does not represent the results of the 2D immunoblot analysis with an opposite pattern. A mixed systemic type 1 and type 2 response in SEA has also been discussed (50–52), which might impact these results and underline the complexity of SEA. Recombinant glucoamylase showed very low overall immunoglobulin binding, and beta-hexosaminidase provoked low IgG3/5 binding in 1D immunoblots suggesting low impact on the binding pattern in 2D immunoblots.

The other four (Xaa-Pro aminopeptidase pepP, Asp f 13, Asp f Catalase, and DPPV) of the eight recombinantly expressed proteins did not reveal significant group differences in Pan-Ig or Ig isotype binding here. Remarkably, probable Xaa-Pro aminopeptidase pepP has also noted protease, metalloprotease, and hydrolase activities like the confirmed antigens. This observation underlines that interesting candidate antigens are not discernible solely based on function, thereby necessitating a comprehensive assessment of protein entities to ascertain their distinct immunogenicity. Due to the expression in *E. coli*, recombinant antigens lack post-translational modifications like phosphorylation or glycosylation, and it cannot be excluded that these would be needed for some Ig binding patterns, hence their absence might impact the observed results.

Alternatively, described major or minor allergens in human asthma might not be central in SEA. For example, Asp f 13 has been observed to cause airway hyperreactivity, T helper cell type 2 responses in mice and to impact remodeling of the airways (53). It was prioritized in the present study based on its description as an allergen but was not confirmed as an antigen in our serological approach here. Similarly, Asp f Catalase is described as a minor allergen and involved in the human pathogenicity of *A. fumigatus* by H_2_O_2_ degradation and protection from reactive oxygen species (54, 55). Moreover, an IgE immunoreactivity in allergic broncho-pulmonary aspergillosis patients has been observed (56). Notably, the expressed *r* catalase here was split into two fragments while peptides covering the whole protein sequence were detected in LC-MS. This might be based on autocatalytic activity and could have influenced antibody binding. The allergen DPPV has been described as a secreted glycoprotein with hydrolase and protease activity and is a known hyphal invasion enzyme (55, 57). Its immunogenicity was detected by human serum IgE and IgG binding from allergic broncho-pulmonary aspergillosis patients (56). However, the overall immunoglobulin binding to Asp f 13, catalase, and DPPV with the horse sera here was less than with the positive control *r*Asp f 1. These three allergens and probable Xaa-Pro aminopeptidase pepP were not confirmed as relevant antigens according to serum Ig binding in our study and accordingly, a simple deduction from human allergic broncho-pulmonary aspergillosis to SEA pathogenesis does not seem applicable.

Ig binding to *A. fumigatus* proteins varied considerably between individuals in contrast to expected similar clustering of sera of horses from the same barn. Accordingly, the environmental matching of asthmatic and healthy horses as pairs with regard to hay exposure and quality was not effective here. Moreover, it is noteworthy that hierarchical clustering exhibited no discernible breed impact on Ig binding. Horses originating from the same barn and of the same breed demonstrated clustering patterns as diverse as those observed among horses from different breeds. In general, a breed predisposition to equine asthma has not been shown (1, 36, 58, 59). Therefore, the present cohort reflects the diagnostic challenge situation of equine patients, which are typically of diverse breeds. Other, intrinsic factors are likely essential in the observed antibody response towards the *A. fumigatus* protein spots (36), but were still strongly impacted by SEA for the immunogenic proteins in the *A. fumigatus* proteome considered relevant antigens here.

Further studies might consider a larger cohort of healthy horses and individuals suffering from SEA to expand conclusions and generate a more comprehensive picture of the immunoreactivity of the candidate antigens suggested here. Due to the nature of the present study, it was not possible to analyze bronchoalveolar lavage cytology or perform lung function testing and respiratory endoscopy on healthy horses matched with asthmatic horses. Therefore, it cannot be fully excluded that horses with mild, subclinical asthma could have been categorized as healthy according to their history (HOARSI) and clinical presentation, while these parameters are highly reliable in detecting SEA (2, 5, 60).

## 5 Conclusion

The process of identifying new candidate antigens by a bottom-up immunoproteomics approach including 2D immunoblot analysis of *A. fumigatus* proteins with specific serum Ig-binding, followed by LC-MS and recombinant expression of selected proteins in *E. coli* seems promising for advancing the comprehension of SEA. Analyses of single *A. fumigatus r* proteins in the present study elucidated antigen specific serum Ig binding from asthmatic horses to unexplored candidate antigens, which have not been considered previously. Additionally, the congruence of certain *A. fumigatus* allergens previously identified for humans with their relevance for SEA-affected horses is not universally evident. Elevated IgG3/5 binding, and decreased IgG4/7 binding to certain proteins emerge as a distinctive facet of SEA. Defining a comprehensive pool of novel candidate antigens forms the basis of more profound insight into the underlying pathogenic mechanisms of specific immune responses in SEA. These can also establish a basis for potential early-stage intervention strategies in the development of this common disease.

## Data availability statement

The datasets presented in this study can be found in online repositories. The names of the repository/repositories (https://panoramaweb.org/Asp-f-in-SEA.url) and accession number(s) can be found in the article/**Supplementary Material**.

## Ethics statement

The animal studies were approved by Veterinary Ethical Committee of all 26 cantons of Switzerland. The studies were conducted in accordance with the local legislation and institutional requirements. Written informed consent was obtained from the owners for the participation of their animals in this study.

## Author contributions

M-CJ: Conceptualization, Data curation, Formal analysis, Investigation, Methodology, Visualization, Writing – original draft, Writing – review & editing. SL: Formal analysis, Investigation, Methodology, Writing – review & editing. WS: Methodology, Resources, Writing – review & editing. DV: Conceptualization, Data curation, Methodology, Resources, Supervision, Writing – review & editing. AK: Methodology, Validation, Writing – review & editing. RH: Conceptualization, Funding acquisition, Methodology, Resources, Writing – review & editing. SK-T: Data curation, Investigation, Resources, Writing – review & editing. VG: Conceptualization, Funding acquisition, Investigation, Resources, Writing – review & editing. EM: Conceptualization, Resources, Writing – review & editing. BW: Data curation, Formal analysis, Investigation, Resources, Writing – review & editing. CS: Conceptualization, Data curation, Funding acquisition, Investigation, Methodology, Project administration, Supervision, Writing – review & editing.

## Supplementary material

Supplementary Figure 1 Preliminary experiments on immunoblots for optimal serum dilutions: **(A–C)** *Aspergillus fumigatus (A. f.)* total protein (TP) preparations, lysate (lys), and recombinant Asp f 1 were separated by PAGE in comparison to a molecular weight marker (MW), blotted, and separate sections of the membranes incubated with different serum dilutions, followed by IgG detection with monoclonal antibodies and fluorochrome-conjugated secondary antibodies. A representative example of IgG4/7 detection is shown (Cy5 detection). With A) serum dilution 1:100 bands at 120 kDa in *A. f.* TP or lys, and at 20 kDa in Asp f 1 resulted in fluorescence saturation of the IgG4/7 binding (highlighted red), while B) serum dilution of 1:250 resulted in distinct detection of several bands within the dynamic range of the fluorescent signal, but C) a serum dilution of 1:500 resulted in loss of signal at weakly detected bands, such as around 15 kDa in *A. f.* lys. A dilution of 1:250 was considered optimal and designated for confirmation on 2D immunoblots. **(D–F)** *Aspergillus fumigatus* protein was separated by 2D electrophoresis (D protein in gel visualized by tryptophan fluorescence, TF), blotted and the membranes incubated with different serum dilutions, followed by the same detection procedure as in A-C. A representative comparison of IgG4/7 detection (Cy5) with the same serum is shown using dilutions of (E) 1:250 or (F) 1:500. Both dilutions resulted in similar saturated spots (red), but with 1:250 more spots were visible here, were detected within the dynamic range of the fluorescence intensities, and could thus be analyzed quantitatively. Lower serum dilutions resulted in more background staining and did not improve spot detection within the dynamic range (not shown). The same strategy was applied for IgG3/5 and Pan-Ig detection, and several different sera were tested before deciding on the final 1:250 dilution for the experimental series.

Supplementary Figure 2 2D immunodetection of asthmatic and healthy horse sera and fused 2D gel image. Paired immunodetection images of asthmatic (red frame) and healthy (black frame) horse sera for immunoglobulins detected (Pan-Ig, Cy3), IgG4/7 and IgG3/5 (Cy5), representative serum samples paired by barn **(A–E)** are displayed. Representative two-dimensional SDS-PAGE (tryptophan fluorescence, fused image) with molecular weights (MW) separated in high (180-65 kDa), medium (65-15 kDa), and low (<15 kDa) molecular weight regions, and spot IDs of all validated protein spots performed in Delta2D 2.8 software (DECODON).

Supplementary Figure 3 Hierarchical clustering after 2D immunodetection of asthmatic and healthy horses does not indicate environmental matching effects. Hierarchical clustering dendrogram of duplicate two-dimensional immunoblots of asthmatic and healthy horse serum (n=5 pairs). Fluorescent intensity values of 289 A*. fumigatus* protein spots of all bound immunoglobulin (Pan-Ig, Cy3), and the isotypes IgG4/7, and IgG3/5 (Cy5) were processed in Delta2D 4.8 software visually representing primarily independent clusters related to environmental matching. Not all spot IDs are shown.
