# Supplementary material for: Immunoproteomics enable broad identification of new Aspergillus fumigatus antigens in severe equine asthma
Source: Front Immunol. 2024 Feb 29;15:1347164. doi: 10.3389/fimmu.2024.1347164 (PMC10937411; doi:10.3389/fimmu.2024.1347164)
Supplement: Supplementary Figure 1 — Preliminary experiments on immunoblots for optimal serum dilutions: (A–C) Aspergillus fumigatus (A. f.) total protein (TP) preparations, lysate (lys), and recombinant Asp f 1 were separated by PAGE in comparison to a molecular weight marker (MW), blotted, and separate sections of the membranes incubated with different serum dilutions, followed by IgG detection with monoclonal antibodies and fluorochrome-conjugated secondary antibodies. A representative example of IgG4/7 detection is shown (Cy5 detection). With A) serum dilution 1:100 bands at 120 kDa in A. f. TP or lys, and at 20 kDa in Asp f 1 resulted in fluorescence saturation of the IgG4/7 binding (highlighted red), while B) serum dilution of 1:250 resulted in distinct detection of several bands within the dynamic range of the fluorescent signal, but C) a serum dilution of 1:500 resulted in loss of signal at weakly detected bands, such as around 15 kDa in A. f. lys. A dilution of 1:250 was considered optimal and designated for confirmation on 2D immunoblots. (D–F) Aspergillus fumigatus protein was separated by 2D electrophoresis (D protein in gel visualized by tryptophan fluorescence, TF), blotted and the membranes incubated with different serum dilutions, followed by the same detection procedure as in A-C. A representative comparison of IgG4/7 detection (Cy5) with the same serum is shown using dilutions of (E) 1:250 or (F) 1:500. Both dilutions resulted in similar saturated spots (red), but with 1:250 more spots were visible here, were detected within the dynamic range of the fluorescence intensities, and could thus be analyzed quantitatively. Lower serum dilutions resulted in more background staining and did not improve spot detection within the dynamic range (not shown). The same strategy was applied for IgG3/5 and Pan-Ig detection, and several different sera were tested before deciding on the final 1:250 dilution for the experimental series. [file DataSheet_1.pdf]

frontiers

Supplementary Material

1 Supplementary Figures and Tables

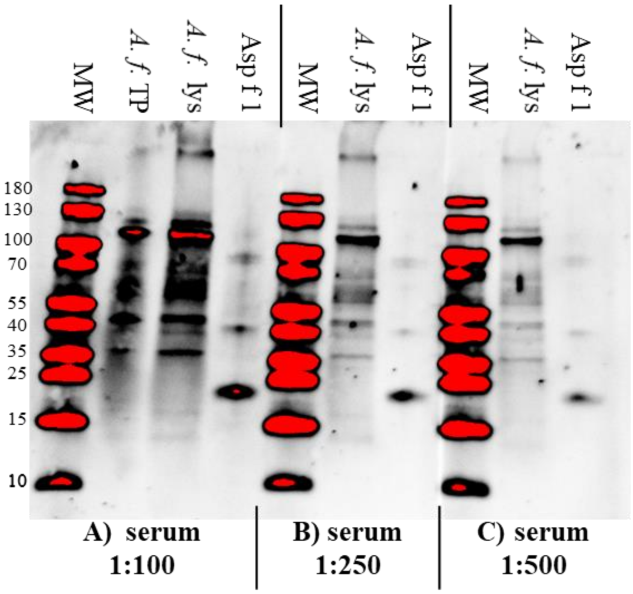

A) serum 1:100 **B) serum 1:250** **C) serum 1:500**

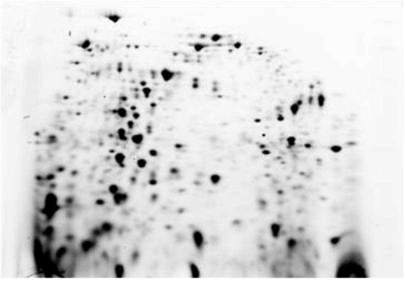

D) *Aspergillus fumigatus* protein (TF)

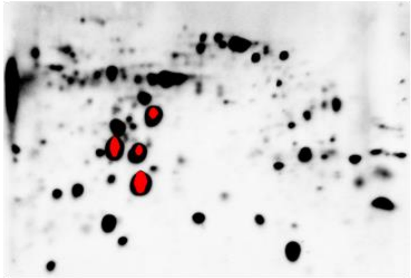

E) serum 1:250

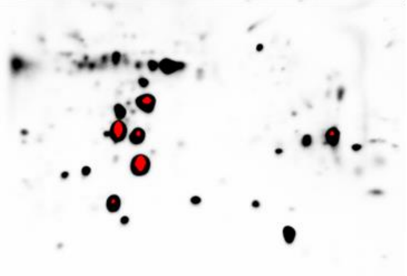

F) serum 1:500

Supplementary Figure 1 Preliminary experiments on immunoblots for optimal serum dilutions**: (A-C)** *Aspergillus fumigatus* (*A. f.*) total protein (TP) preparations, lysate (lys), and recombinant Asp f 1 were separated by PAGE in comparison to a molecular weight marker (MW), blotted, and separate sections of the membranes incubated with different serum dilutions, followed by IgG detection with monoclonal antibodies and fluorochrome-conjugated secondary antibodies. A representative example of IgG4/7 detection is shown (Cy5 detection). With A) serum dilution 1:100 bands at 120 kDa in *A. f.* TP or lys, and at 20 kDa in Asp f 1 resulted in fluorescence saturation of the IgG4/7 binding (highlighted red), while B) serum dilution of 1:250 resulted in distinct detection of several bands within the dynamic range of the fluorescent signal, but C) a serum dilution of 1:500 resulted in loss of signal at weakly detected bands, such as around 15 kDa in *A. f.* lys. A dilution of 1:250 was considered optimal and designated for confirmation on 2D immunoblots. **(D-F)** *Aspergillus fumigatus* protein was separated by 2D electrophoresis (D protein in gel visualized by tryptophan fluorescence, TF), blotted and the membranes incubated with different serum dilutions, followed by the same detection procedure as in A-C. A representative comparison of IgG4/7 detection (Cy5) with the same serum is shown using dilutions of (E) 1:250 or (F) 1:500. Both dilutions resulted in similar saturated spots (red), but with 1:250 more spots were visible here, were detected within the dynamic range of the fluorescence intensities, and could thus be analyzed quantitatively. Lower serum dilutions resulted in more background staining and did not improve spot detection within the dynamic range (not shown). The same strategy was applied for IgG3/5 and Pan-Ig detection, and several different sera were tested before deciding on the final 1:250 dilution for the experimental series.

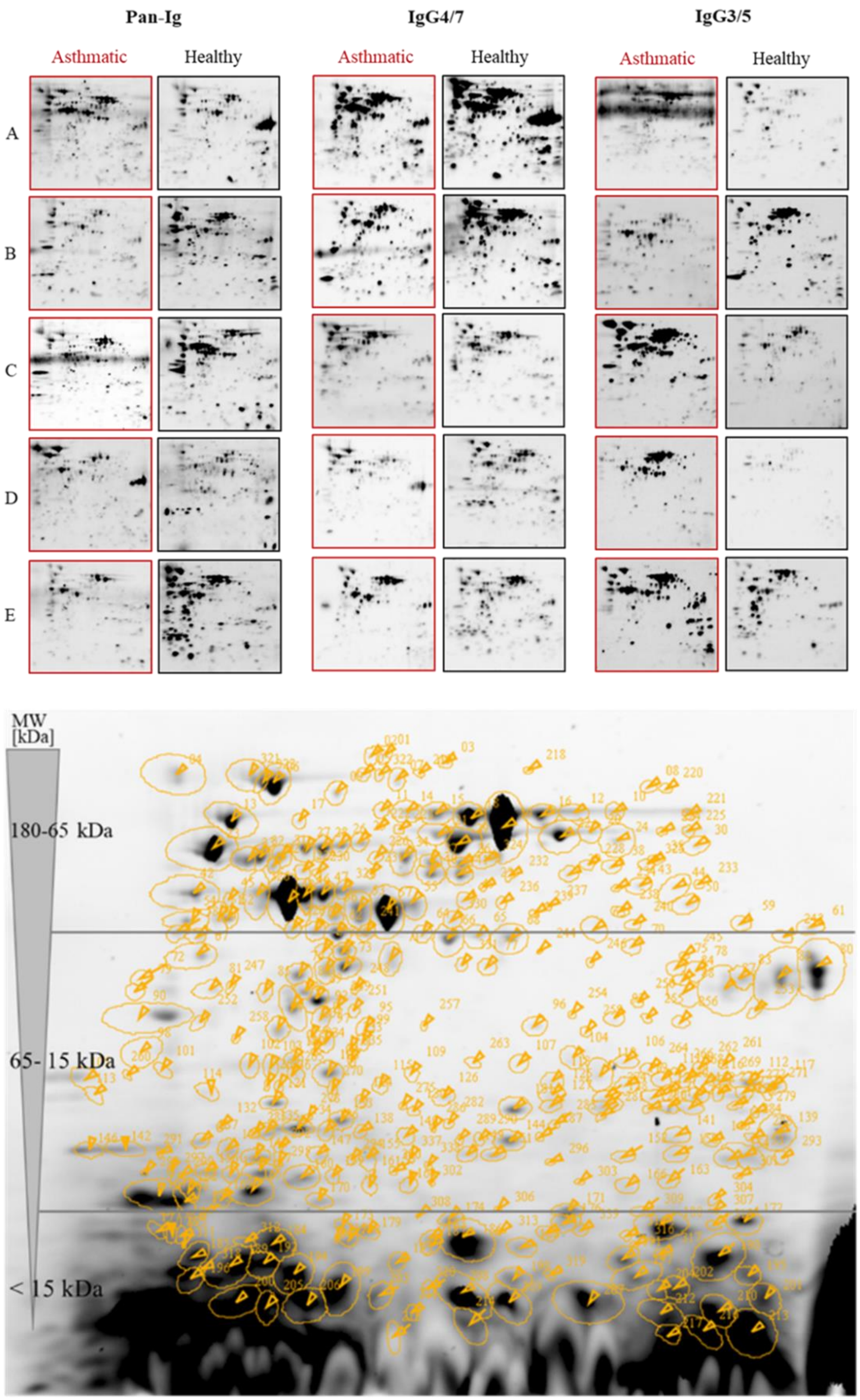

Supplementary Figure 2 2D immunodetection of asthmatic and healthy horse and fused 2D gel image

Paired immunodetection images of asthmatic (red frame) and healthy (black frame) horse sera for immunoglobulins detected (Pan-Ig, Cy3), IgG4/7 and IgG3/5 (Cy5), representative serum samples paired by barn (A–E) are displayed.

Representative two-dimensional SDS-PAGE (tryptophan fluorescence, fused image) with molecular weights (MW) separated in high (180-65 kDa), medium (65-15 kDa), and low (<15 kDa) molecular weight regions, and spot IDs of all validated protein spots performed in Delta2D 2.8 software (DECODON).

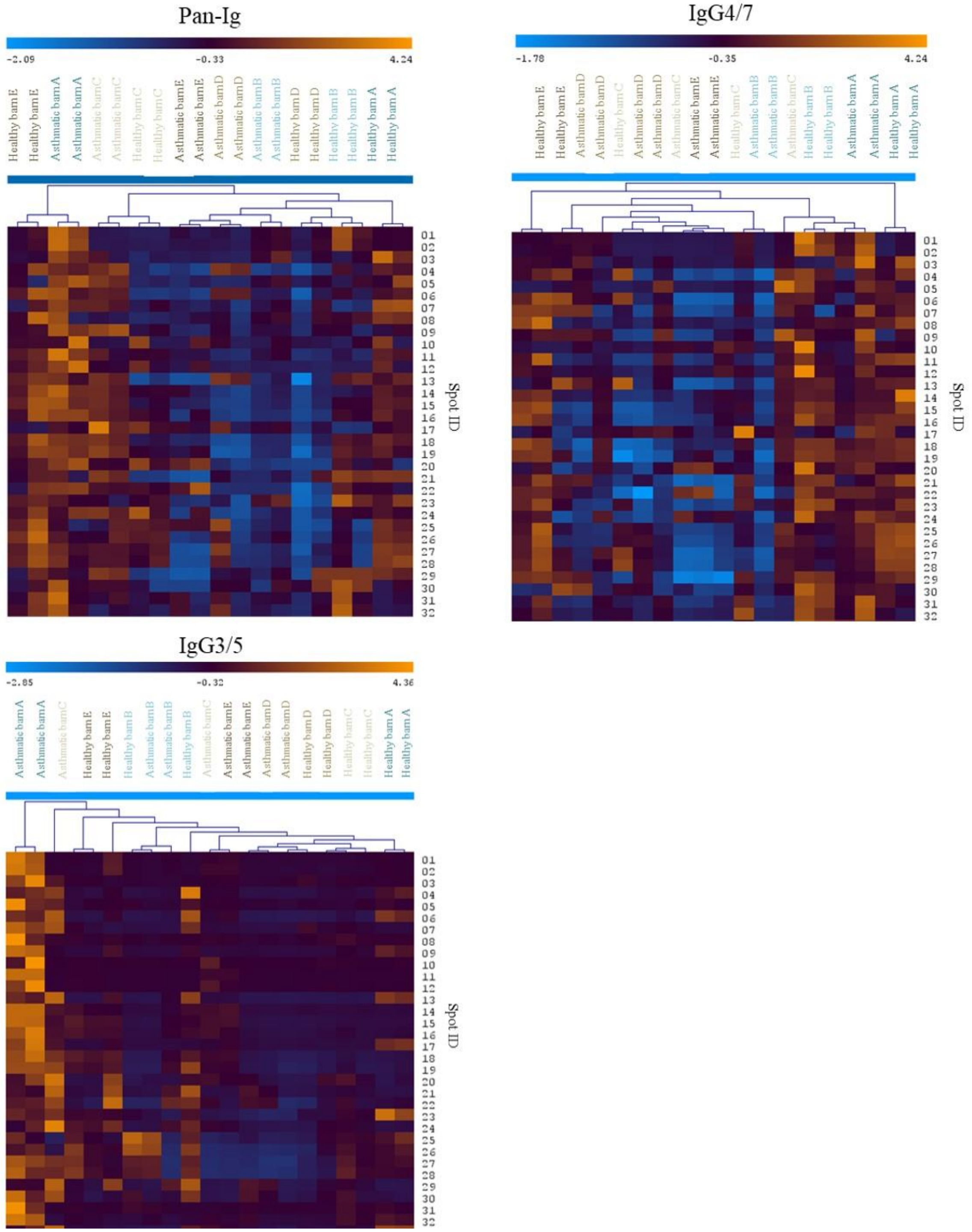

Supplementary Figure 3 Hierarchical clustering after 2D immunodetection of asthmatic and healthy horses does not indicate environmental matching effects
Hierarchical clustering dendrogram of duplicate two-dimensional immunoblots of asthmatic and healthy horse serum (n=5 pairs). Fluorescent intensity values of 289 *A. fumigatus* protein spots of all bound immunoglobulin (Pan-Ig, Cy3), and the isotypes IgG4/7, and IgG3/5 (Cy5) were processed in Delta2D 4.8 software visually representing primarily independent clusters related to environmental matching. Not all spot IDs are shown.

Supplementary Tables

Supplementary Table 1 Target gene amplifications with primer, and PCR conditions of finally unexpressed *A. fumigatus* proteins

Target proteins	Uniprot	Forward primer (restriction site)[a–e]	Reverse primer (restriction site)[a–e]	Annealing temperature	Elonga-tion time
Alpha-galactosidase	B0YDJ1	*CGCTTACATATGATGCATATCTCGGGCTATAAAATC*[a]	*GCCATCCTCGAGCAATTCATCCTTTTTGTCGGTCG*[b]	55 °C	80 sec
Beta-fructo-furanosidase, putative	B0XT79	*GCAATTCATATGATGGCCGCGGTAGATGCAAT*[a]	*GCGAATGCGGCCGCGGATGCAGCTAGTCTCTCTAG*[c]	55 °C	40 sec
Extracellular serine-rich protein, putative	B0XYK6	*CGCATTCATATGATGGTCTTCGCGTGGGTGA*[a]	*CGAATTAAGCTTCAAAGCAATAGGAGTGGAAAGC*[d]	55 °C	100 sec
Glutaminase GtaA	B0XYT5	*CGGCATCATATGGTGTCAACTTTCTCTCCTGCA*[a]	*GCGAATGCGGCCGCAAGACCCTCCTCCAGAAGTAAT*[c]	55 °C	80 sec
Isopentenyl-diphosphate Delta-isomerase	B0Y8F7	*GCTTGACATATGATGACATCCACCGCAACAGTTA*[a]	*GCTAAACTCGAGCATGCGGCGAATCTCCTTCT*[b]	55 °C	40 sec
MFS peptide transporter, putative	B0YAB1	*CGGCATCATATGAACGTCTCGGACCCCGTC*[a]	*GCGAAAGCGGCCGCCTGCCGCACGGACTCCT*[c]	55 °C	80 sec
Neutral ceramidase	B0XPL9	*CGGCATCATATGGCAAACTCCAGACTCGGAGT*[a]	*GCTATTGCGGCCGCGCCCAAATTCACCGTGAATGG*[c]	56 °C	80 sec
Probable alpha/beta-glucosidase agdC	B0XNL6	*CGAATTCATATGGCCGTGATCGGCGCCA*[a]	*CGAATTAAGCTTGCTGAGGTCAATCTCGGAAG*[d]	54°C	100 sec
Probable beta-glucosidase A	B0XPE1	*CGGCATGGATCCATGAGATTCGGTTGGCTCGAG*[e]	*CGGCATAAGCTTGTAGACACGGGGCAGAGG*[d]	56 °C	100 sec
Probable mannosyl-oligosaccharide alpha-1,2-mannosidase 1B	B0XMT4	*GCTTTACATATGATGCATTTACCCTCTTTGTCCGT*[a]	*GCTTATCTCGAGTCCTCTGCTCTTGTTAGCTTTAT*[b]	56 °C	80 sec
Telomere and ribosome associated protein Stm1, putative	B0XXQ3	*CGGCATCATATGATGGCGGACGTCCGGTC*[a]	*GCTATTGCGGCCGCTTATTTGGCACCGAGGGAGG*[c]	55 °C	40 Sec

Restriction sites for (underlined): [a]Ndel, [b]Xhol, [c]Notl, [d]HindIII, [e]BamHl
